# Promoting Simultaneous Onset of Viral Gene Expression Among Cells Infected with Herpes Simplex Virus-1

**DOI:** 10.3389/fmicb.2017.02152

**Published:** 2017-11-01

**Authors:** Maya Ralph, Marina Bednarchik, Enosh Tomer, Dor Rafael, Sefi Zargarian, Motti Gerlic, Oren Kobiler

**Affiliations:** Department of Clinical Microbiology and Immunology, Sackler School of Medicine, Tel Aviv University, Tel Aviv, Israel

**Keywords:** single cell, biological noise, timing of infection, temperature sensitive, herpesviruses

## Abstract

Synchronous viral infection facilitates the study of viral gene expression, viral host interactions, and viral replication processes. However, the protocols for achieving synchronous infections were hardly ever tested in proper temporal resolution at the single-cell level. We set up a fluorescence-based, time lapse microscopy assay to study sources of variability in the timing of gene expression during herpes simplex virus-1 (HSV-1) infection. We found that with the common protocol, the onset of gene expression within different cells can vary by more than 3 h. We showed that simultaneous viral genome entry to the nucleus can be achieved with a derivative of the previously characterized temperature sensitive mutant tsB7, however, this did not improve gene expression synchrony. We found that elevating the temperature in which the infection is done and increasing the multiplicity of infection (MOI) significantly promoted simultaneous onset of viral gene expression among infected cells. Further, elevated temperature result in a decrease in the coefficient of variation (a standardized measure of dispersion) of viral replication compartments (RCs) sizes among cells as well as a slight increment of viral late gene expression synchrony. We conclude that simultaneous viral gene expression can be improved by simple modifications to the infection process and may reduce the effect of single-cell variability on population-based assays.

## Introduction

Diversity among genetically identical cells during viral infection leads to significant heterogeneity in the outcome of infection. This can be detected as large differences in viral yield per cell (Delbruck, 1945; Zhu et al., 2009; Timm and Yin, 2012; Heldt et al., 2015), in viral genetic diversity (Combe et al., 2015; Cohen and Kobiler, 2016) and in the timing of different events in the infection process (Amir et al., 2007; Timm and Yin, 2012; Akpinar et al., 2015; Drayman et al., 2017). Several studies suggest that cell conditions (such as cell cycle, specific genes expression levels, and cell density) prior to infection play a significant role in determining the outcome of infection (Snijder et al., 2009; Cohen and Kobiler, 2016; Drayman et al., 2017). In recent years, thanks to advancements in technology, the study of viral infection at the single-cell level has allowed a better resolution for studying viral host interactions (even in the well-studied case of bacteriophage lambda Golding, 2016).

Cell-to-cell diversity during viral infection can impair the interpretation of traditional population-based genetics and biochemistry assays. Therefore, it is a common practice in virology to try to reduce the variability among cells during infection for these types of assays. The first approach is to ensure that all cells will be infected with at least one viral particle. The most common method to achieve this goal is to infect using a high multiplicity of infection (MOI) (Condit, 2013). Alternative methods, like spinoculation (Tenser and Dunstan, 1980) and magnetically controlled virus inoculation (Haim et al., 2005) also increase the probability of viruses to infect cells. The second approach is to “pause” the infection, by inhibiting a specific step during the infection process and simultaneously removing the inhibiting factor from all cells. The most common method to achieve this requires the inhibition of the virion fusion by incubating at a low temperature (0–4°C) followed by a rapid shift to 37°C to allow fusion at the same time (Bedson and Gostling, 1956; Farnham and Newton, 1959). A different approach is to synchronize the population of cells to a specific point during cellular growth and division. This is usually done by arresting the cell in a specific cell stage, either by using specific inhibitors or by starvation. Remarkably, for herpesviruses these approaches were not compared in a single-cell assay in detailed kinetic resolution.

**Figure 1:**
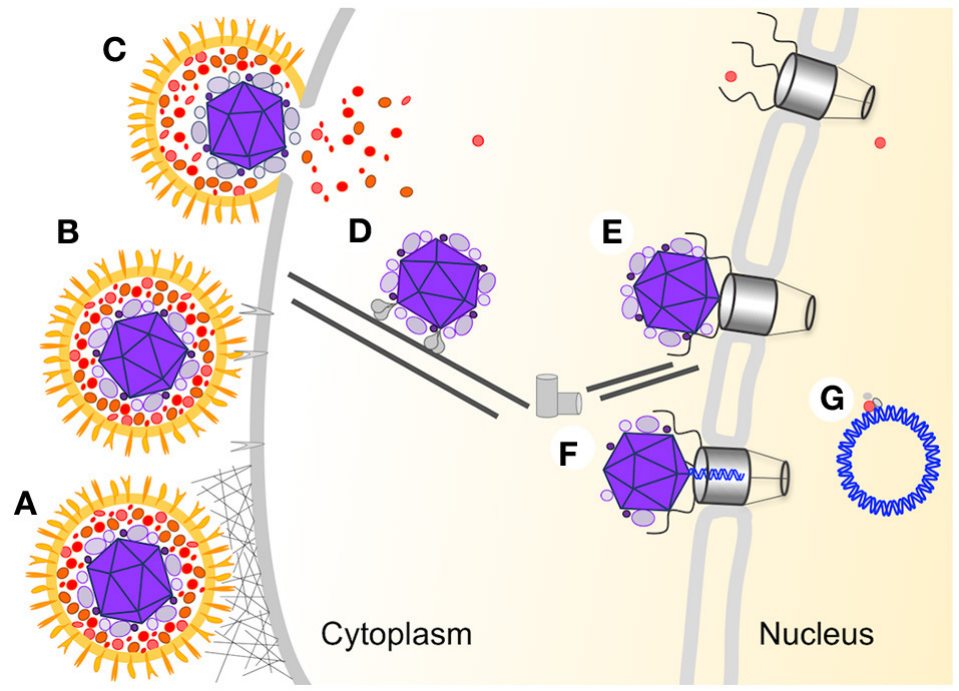
HSV-1 multistep entry process. A schematic illustration of the different steps involved in the entry of HSV-1 into the host nucleus. The viral particle is colored orange (membrane and membrane protein), red (outer tegument layer), and purple (inner tegument and capsid), the viral DNA is in blue and all host proteins and structures are in different shades of gray. **(A)** Non-specific attachment between HSV-1 glycoproteins with heparan sulfate on the cell surface. **(B)** Specific binding between gD and the host specific receptors. **(C)** Fusion between the viral and host membrane. **(D)** Transport of the capsid to the nucleus is mediated by the viral inner tegument proteins interaction with cellular microtubule motor proteins. **(E)** Docking of the capsid to the nuclear pore complex (NPC). **(F)** Following conformational change in the capsid the viral DNA is translocated into the nucleus through the NPC. **(G)** The viral DNA adopt a circular conformation within the nucleus and viral gene expression is initiated.

Herpesviruses belong to a diverse family of large, double-stranded DNA viruses that replicate in the nucleus of infected cells. Herpes simplex virus-1 (HSV-1) is a prototypical model system to study of elements of viral infection and replication common to all members of the Herpesviridae. The structural features of the viral particles are conserved among members of the family. The linear DNA is tightly packed within a capsid shell (Bauer et al., 2013). The capsid is surrounded by the tegument, an amorphous layer of viral and host proteins (Loret et al., 2008; Stegen et al., 2013). The outer layer is the viral membrane which contains several different viral glycoproteins. The entry process (illustrated in Figure 1) is initiated when a free virion interacts with the host membrane and is concluded when the viral DNA begins to be transcribed in the nucleus. This multistep process (recently reviewed in Agelidis and Shukla, 2015; Kilcher and Mercer, 2015) allows the shedding of the viral surrounding layers (illustrated in Figure 1). Here, we compared the results of altering different steps in the entry process on synchronous gene expression from the viral genomes.

One of the key steps of the entry process is the release of the viral genome from the viral capsid and into the nucleus through the nuclear pore complex (NPC) (recently reviewed by Kobiler et al., 2012; Fay and Pante, 2015). The viral capsid and some of the inner tegument proteins arrive at the nuclear membrane and dock to the NPC (Ojala et al., 2000). Following docking to the NPC, the capsid undergoes a conformational change which allows the release of the viral DNA and its translocation into the nucleus. The mechanisms initiating conformational change to the capsid are yet to be identified. However, the involvement of several viral proteins in this process has been established. After capsid docking to the NPC, the large tegument protein VP1/2 (encoded by the UL36 gene) must be cleaved to allow the viral genome to be released into the nucleus (Jovasevic et al., 2008). Further evidence of the role of VP1/2 in this process comes from the identification of a temperature-sensitive (ts) mutation (tsB7) mapped to the UL36 gene that allows docking to the NPC but prevents DNA release into the nucleus at a non-permissive temperature (NPT) (Batterson et al., 1983). This tsB7 mutation was characterized as a single amino acid change, Y1453H, in the VP1/2 protein (Abaitua et al., 2011).

HSV-1 cause a rapid lytic infection: gene expression can be detected almost 1 h post infection (HPI), progeny viruses can be detected at 6 HPI and most of the infected cells are dead by 16–24 HPI. Here, we monitor viral gene expression for the first 10 h following HSV-1 infection at the single-cell level using a recombinant virus expressing a fluorescent reporter protein. We compare the relative synchrony in timing of gene expression onset among different conditions of infection. Our results suggest that common practices in cell-culture viral infection might not be effective in synchronizing the infection. On the other hand, all mechanisms that increase the progression rate of infection reduce the variability among cells. Further, our results support the hypothesis that the major source of variation among cells stems from viral host interactions within the host nucleus.

## Results

### Diversity in the onset time of gene expression from viral genomes among cells

**Figure 2:**
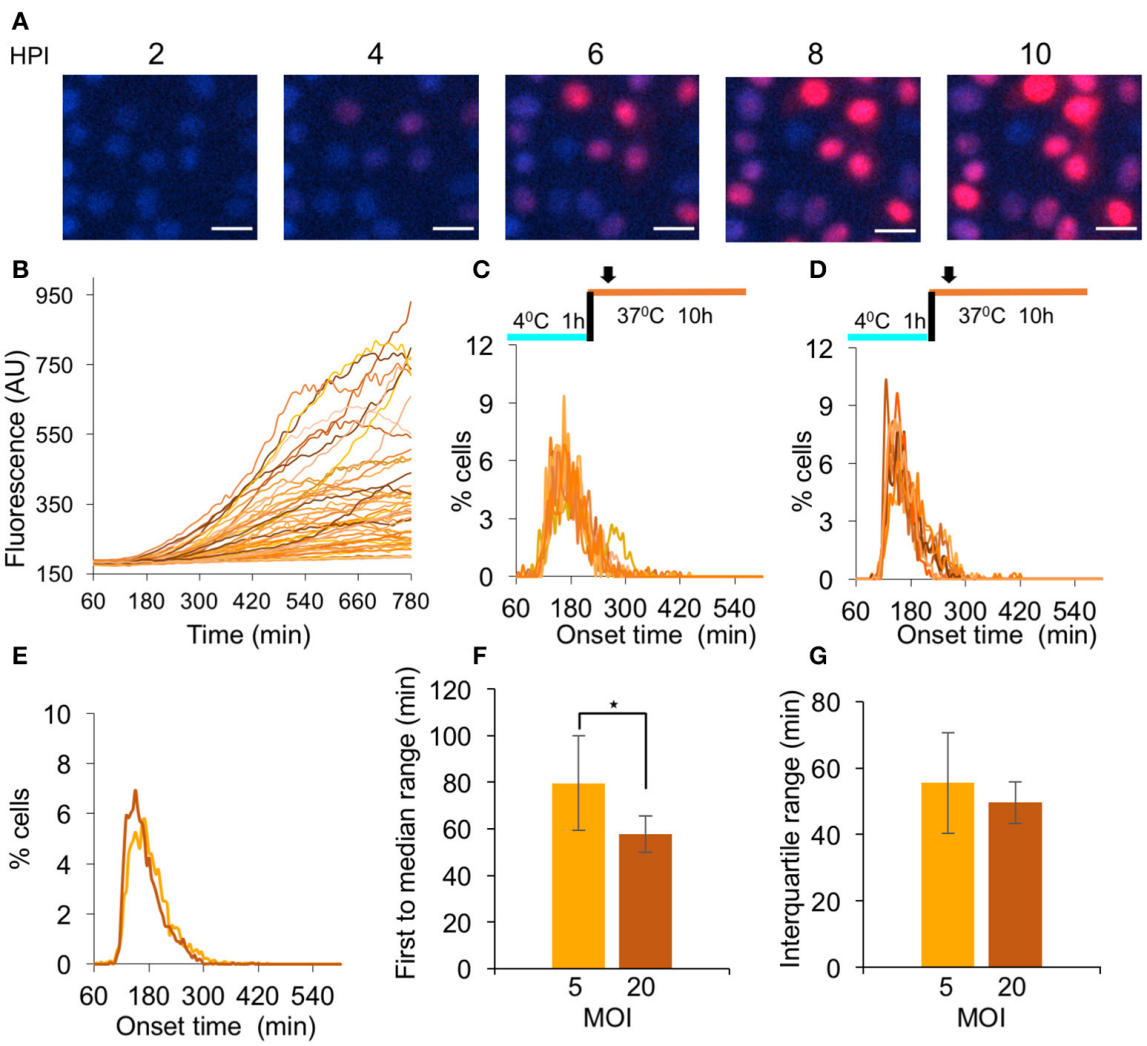
Analysis of HSV-1 simultaneous gene expression. **(A–C)** Cells infected with OK11 recombinant at MOI 5 were monitored for 12 h. **(A)** Snapshots of cells at different time points (as indicated) are presented. Nuclei are colored with Hoechst stain in blue, mCherry fluorescence in red. Scale bar 20 μm. **(B)** The red fluorescence profiles in arbitrary units (AU) for individual cells during the infection were plotted. **(C)** Each histogram presents the percent of cells at each time point, that initiated expression (binning every 5 min) in a single well; eight wells from two experiments done on different days were plotted. **(D)** Same histograms as C for cells infected with OK11 recombinant at MOI 20. **(C–D)** The infection conditions are schematically illustrated above each graph. Arrows represent imaging starting time (~1 h from last temperature shift). **(E–G)** Comparison between the onset times of expression for infections at MOI 5 (light orange) and MOI 20 (dark orange). **(E)** The average histograms for each condition were plotted. **(F)** The time between the first cell onset of expression to the median cell onset (first to median range) were plotted for each condition. **(G)** The interquartile range was plotted for each condition. **(F–G)** Error bars represent the standard deviation among the different wells. ^*^P < 0.05 by student *T*-test.

To achieve synchronous HSV-1 infection, one usually infects under the following conditions: all cells are maintained in G1/G0 state; infection is done with a high MOI (above 3) and absorption of the viruses is carried out at 4°C for 1 h. To test if this method indeed provides a simultaneous viral gene expression, we infected Vero cells, grown in serum depleted medium for 48 h prior to infection, with a virus encoding the mCherry fluorescent protein (HSV-1 OK11) at MOI 5 and monitored the fluorescence accumulation in each infected cell using time-lapse microscopy (Supplementary Movie 1 and Figure 2A). Our results indicate that under these conditions, there are differences of more than 3 h among infected cells with regard to the onset of the red fluorescence. To quantify these results and compare different conditions, we plotted the fluorescence level from each cell at each time point (Figure 2B). We developed a method to estimate the time of fluorescence onset for each cell (see section Methods and Supplementary Figure 1) and found that the detectable fluorescence could be measured as early as 68 min after the temperature shift (4–37°C). However, most cell fluorescence onsets were detected between 100 and 300 min (Figure 2C). These results indicate that common infection practice does not yield synchronized infection.

To test whether a higher MOI could increase the simultaneous onset of gene expression from the viral genomes, we repeated the experiments at MOI 20 (Figure 2D). We compared the average histograms obtained from merging all the histograms from each condition (MOI 5 and 20, Figure 2E) and observed that at MOI 20, a slightly higher percentage of the cells initiated gene expression in a shorter time period. To test if this difference is significant, we used two parameters that provide a good estimate of the simultaneous onset of viral gene expression by the majority of the infected cells. We measured the time from the first cell onset to the median cell onset (i.e., the first 50% of the cells; first to median range). This is probably the most intuitive parameter for describing simultaneous gene expression, however it is highly influenced by very early individual cells. We therefore also measured the interquartile range, the time from the onset of the 25-percentile cell to the onset of the 75-percentile (i.e., middle 50% of the cells). We compared the first to median range, and found that at MOI 20, significantly less time was needed for half of the cells to initiate expression (39.3 compared to 72.4 min, *P* = 0.026 Figure 2F). We further compared the time for the interquartile range and found only a small difference between the MOIs (49.6 compared to 55.5 min, Figure 2G). These results indicate that a higher MOI can improve the simultaneous onset of viral gene expression, however, even at MOI 20, the variability in onset time is substantial.

### Synchronized nuclear entry does not increase simultaneous gene expression

**Figure 3:**
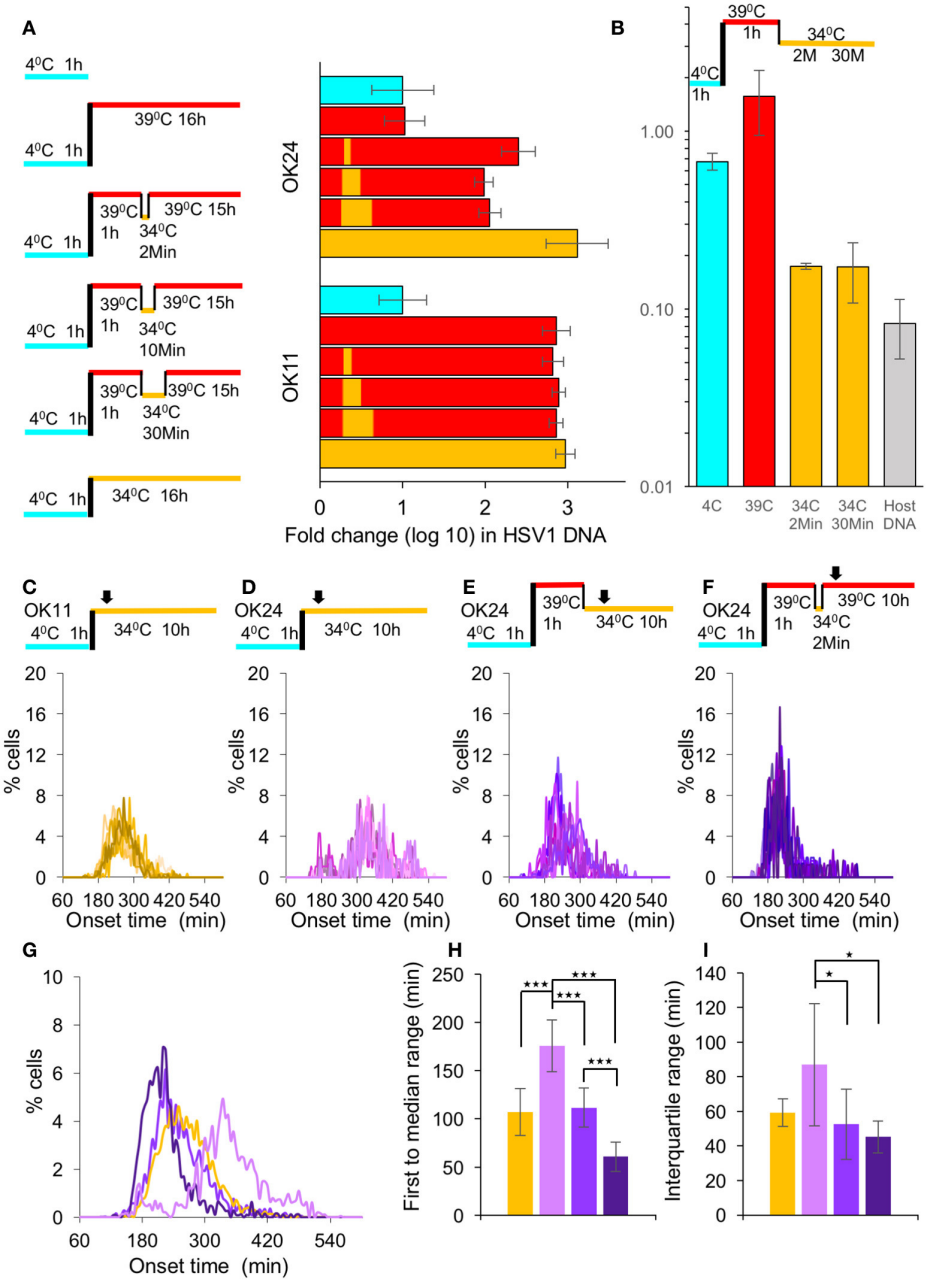
Synchronous nuclear entry does not increase simultaneous gene expression. **(A)** Left panel—A schematic representation of the six different temperature conditions (On ice—light blue, 34°C—yellow and 39°C—red) in which the viral DNA levels were measured. Right graph—Cells were infected with either OK11 or OK24 fold-change differences of viral DNA levels between the different conditions (color-coded as the left panel), as measured by qPCR; each condition was repeated three times. Error bars represent the standard error. **(B)** Cells were infected with ts viral recombinant at MOI 20. At different stages of the infection conditions of infection are illustrated above the graph, color coded as **(A)**, the cells were collected. The amount of capsid protected viral DNA in the cells divided by the total amount of viral DNA at each point are presented. An average of two technical replicates (each measured by qPCR). Error bars represent the standard error. **(C–F)** All infections were done at MOI 20. Histograms present the percentage of cells at each time point, that initiated expression (binning every 5 min). At least eight wells from at least two experiments done on different days were plotted. The infection conditions are schematically illustrated above each graph. Arrows represent imaging starting time (~1 h from last temperature shift). **(C)** Cells were infected with OK11 recombinant virus at 34°C. **(D)** Cells were infected with OK24 recombinant virus at 34°C. **(E)** Cells were infected with OK24 recombinant virus at 39°C for 1 h and shifted to 34°C for the rest of the experiment. **(F)** Cells were infected with OK24 recombinant virus at 39°C for 1 h and shifted to 34°C for 2 min. **(G–I)** A comparison between the onset times of expression for the infection presented in **(C)** (yellow), **(D)** (light purple), **(E)** (purple), and **(F)** (dark purple). **(G)** The average histograms for each condition were plotted. **(H)** The time between the first cell onset of expression to the median cell onset (first to median range) was plotted for each condition. **(I)** The interquartile range was plotted for each condition. **(G–I)** Error bars represent the standard deviation among the different wells. ^*^P < 0.05, ^***^P < 0.001 by student *T*-test.

For herpesviruses, both transcription and replication occur in the nucleus of the infected cell. We therefore hypothesize that movement toward the nucleus and nuclear entry might be an important source for variability in the onset of viral gene expression. To test this hypothesis, we decided to monitor the expression during infection with a temperature sensitive (ts) mutant virus that is unable to enter the nucleus at the NPT. By homologous recombination we obtained an HSV-1 strain carrying the single amino acid change, Y1453H, in the VP1/2 protein (that results in the ts phenomenon) and encoding the mCherry fluorescent protein (HSV-1 OK24). Next, we wanted to demonstrate that the temperature shift can lead to a rapid entry into the nucleus. Cells were infected with either OK11 or OK24 and the viral DNA levels were measured at 16 HPI and compared to the input DNA levels (measured after the 1 h incubation at 4°C). We tested five different infection conditions; incubation at the NPT (39°C), incubation at the NPT for 1 h, rapid shifting to the permissive temperature (PT 34°C) for 2, 10, or 30 min and shifting back to 39°C, and infection at the PT for all 16 h, as illustrated in Figure 3A. As expected, the ts recombinant could not initiate replication of viral DNA, if it remained in the NPT for the entire incubation time. However, even the shortest shift to 34°C (i.e., 2 min) was sufficient to result in viral DNA replication. Interestingly, longer time at the PT (up to 30 min) did not seem to increase viral DNA replication, suggesting that the short time allows most of the available viral genomes to enter the nucleus. We noted that when the ts recombinant was kept at the PT for the entire infection, viral replication was higher compared to when infection was shifted back to the NPT (Figure 3A). These results support previous findings, suggesting that this ts mutation has other effects on the viral replication cycle in addition to nuclear entry (Abaitua et al., 2009). The different temperature conditions tested for the OK24 recombinant were also tested on the OK11 (non-ts) recombinant. In the case of OK11, infection at all temperature conditions results in similar levels of viral DNA replication without significant differences, suggesting that the differences observed in the OK24 recombinant are due to the ts phenomenon. We conclude that the ts recombinant (OK24) can enter the nucleus only at the PT and require only 2 min at the PT.

Our results indicate that 2 min at the PT are sufficient to allow the virus to enter the cells, probably by initiating the conformational change needed for viral genome release. To assume a synchronous nuclear entry, we tested if the viral genomes are released from the capsids within the short time at the PT. We incubated cells with the ts virus for 1 h at 4°C, then transferred the cells to 39°C for additional hour. After rapid shift of temperature, we collected the cells either at 2 or 30 min after the temperature shift. The collected cells at the different stages of the infection, were divided to two population, with or without DNase I. For each condition, we compared by qPCR the amount of viral DNA that was protected from the DNAse. Our results (Figure 3B) indicate that at 4 or 39°C, most of the viral DNA was protected from degradation by the DNase, however once the cells were expose to the PT even for 2 min, most of the viral DNA (over 80%) was degraded. Host DNA under the same conditions was more than 90% degraded. These results indicate that the rapid shift in temperature from the NPT to the PT results in rapid release of the viral DNA from the capsid and indicate synchronous nuclear entry.

To test if the synchronous nuclear entry can reduce the variability of the onset of gene expression from the viral genome, we compared the infection with OK11 (Figure 3C) to OK24 (Figures 3D–F). First, we infected with both viruses at the PT (34°C) and monitored the fluorescence levels. The results indicate that the ts virus (OK24) is slower (median time was 343.2 min for OK24 and 262.9 min for OK11) and has higher variability compared to the wild type virus (Figures 3C,D,G). This difference was mostly observed by measuring the time of first to median range (107.2 compared to 175.9 min, *P* < 0.001, Figure 3H). As observed with the differences in MOI, the interquartile range was not significant (59.4 compared to 86.9 min, *P* = 0.064, Figure 3I). These results suggest that, even at the PT, the mutant virus probably has slower kinetics in reaching and entering the nucleus (as one would expect from a VP1/2 mutant; Abaitua et al., 2009; Schipke et al., 2012; Zaichick et al., 2013). To test the effect of synchronous nuclear entry, following the absorption of the ts virus into the cells at 4°C, the cells were transferred a NPT (39°C) for 1 h, and then underwent a rapid shift to the PT (Figure 3E). Under these conditions (continuous PT), the speed of onset increased (median time was 250.0) and variability decreased (compared to the OK24 infection at 34°C) to levels similar to OK11 infection at 34°C (Figure 3G). Both the first to median range as well as the interquartile range were significantly shorter between the infection with OK24 continuous PT and OK24 infection at 34°C (*P* = 0.00012 and 0.035, Figure 3H,**I**, respectively). The OK24 results suggest that the accumulation of viral capsids on the nuclear membrane followed by synchronous genome entry may reduce the variability in viral gene expression among cells. However, a comparison of the OK24 continuous PT experiment with the OK11 infection at 34°C showed no significant difference, suggesting that the OK24 continuous PT overcomes the defect of the ts mutant but does not improve variability in gene expression when compared to wild type infection.

Our results suggest that only 2 min at the PT are sufficient to allow most of the viral genomes to enter the cells. We hypothesize that by limiting the possible entry time to 2 min, we can increase the simultaneous onset of viral gene expression. We therefore incubated the ts virus with the cells at 4°C for 1 h and then the transferred the cells to NPT (39°C) for 1 h. We then performed a rapid shift to the PT for 2 min and then immediately shifted them back to 39°C for the rest of the experiment (Figure 3F). Under these conditions (2 min PT), the speed of onset increased (median time was 211.8) and variability decreased, in comparison to the experiment performed with continuous PT or OK11 infection at 34°C experiments (Figures 3G). The first to median range and the interquartile range of the 2 min PT infection were significantly shorter than OK11 or OK24 infection at 34°C (Figures 3H,I). However, when comparing the 2 min PT infection to continuous PT infection, the first to median range was significantly shorter (60.7 compared to 111.8 min, *P* < 0.001, Figure 3H) but the interquartile range was not significantly different (45.3 compared to 52.5 min, *P* = 0.372, Figure 3I). These results suggest that limited time at the PT provide a more synchronous infection.

### Elevated temperature increases simultaneous gene expression

**Figure 4:**
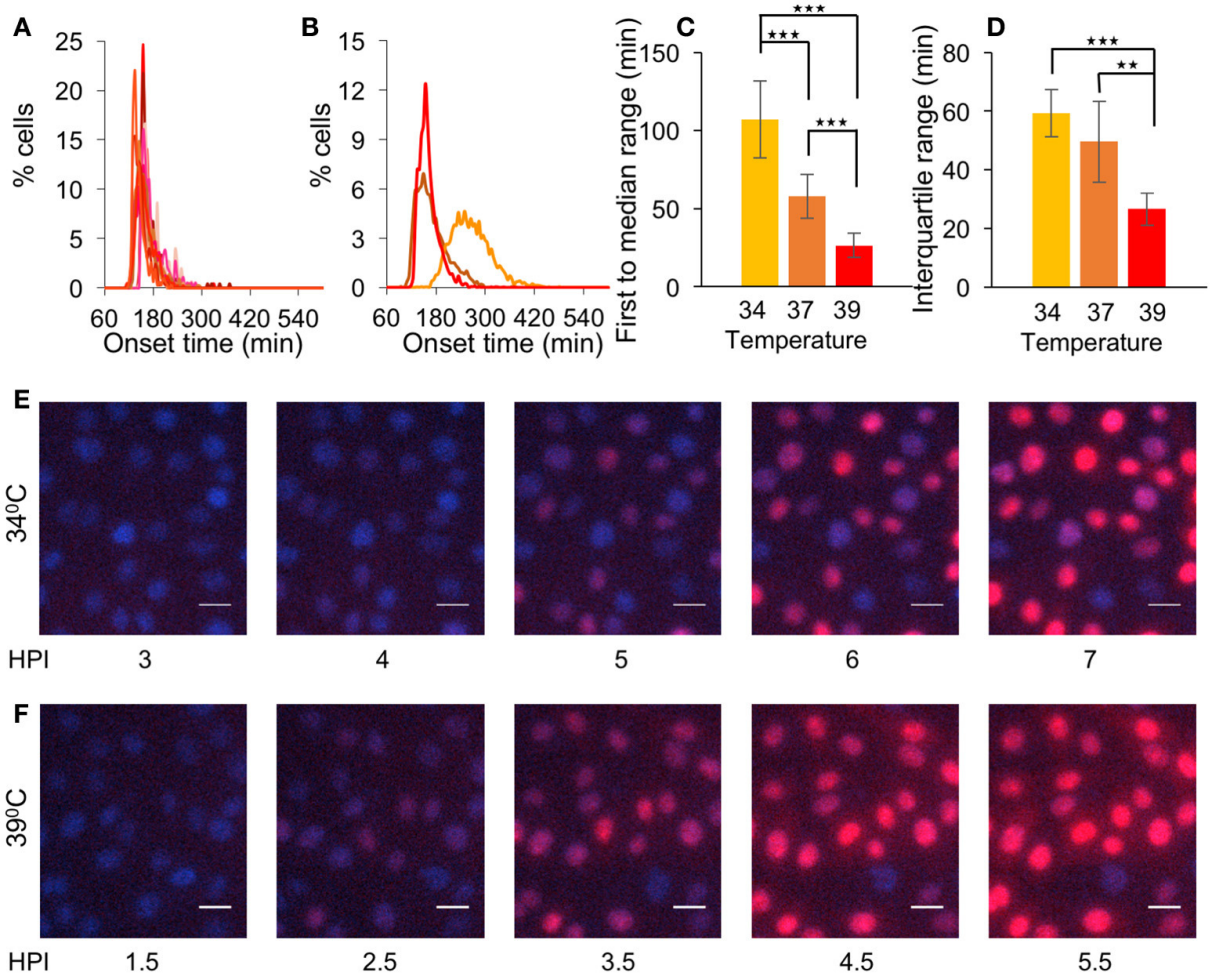
Elevated temperature increases simultaneous gene expression. **(A)** Cells were infected with OK11 recombinant virus at 39°C, at MOI 20. Histograms present the percentage of cells at each time point, that initiated expression (binning every 5 min). Fourteen wells from four experiments done on different days were plotted (at least two wells per experiment). **(B–D)** A comparison between the onset times of expression for the infection at different temperatures: 34°C (yellow, data from Figure 3B), 37°C (orange, data from Figure 2D) and 39°C (red, data from **(A)**. **(B)** The average histograms for each condition were plotted. **(C)** The time between the first cell onset of expression to the median cell onset (first to median range) was plotted for each condition. **(D)** The interquartile range was plotted for each condition. **(B–D)** Error bars represent the standard deviation among the different wells. ^**^P < 0.01, ^***^P < 0.001 by student *T*-test. **(E–F)** Cells were infected with OK11 recombinant virus at MOI 20 and incubated at either 34°C **(E)** or 39°C **(F)**. Snapshots at different time points (as indicated) are presented. Nuclei are colored with Hoechst stain in blue, mCherry fluorescence in red. Scale bar 20 μm.

To test whether the increased simultaneous gene expression in the 2 min PT infection experiment is due to the short time allowed for nuclear entry or to the higher temperature at which the experiment was done, we monitored OK11 infection at 39°C. The histograms of the onset time show a much sharper curve (Figure 4A) compared to that of previous experiments, indicating simultaneous gene expression. Indeed, when comparing the 39–37°C or 34°C (comparison of Figures 2D, 3C, 4A summarized in Figure 4B as merged histograms), both 39 and 37°C have a similar onset time (median time 158.7 and 165.3 min, respectively), which is faster than with infection at 34°C (median time 262.9 min). However, only with 39°C does the peak of the histogram (binning every 5 min) reach more than 10% of cells, indicating that many cells initiate viral gene expression within a short period of time. When comparing the first to median range and the interquartile range at the three temperatures (Figures 4C,D, respectively), it can be clearly seen that the ranges shorten as the temperature raises (*P* < 0.002 for all comparisons with the exception of the interquartile range between 34 and 37°C which is not statistically significant). This difference is significant enough to be detected even without computational analysis (Supplementary Movies 2, 3 and Figures 4E,F). These results suggest that different temperatures can significantly change the synchrony of the infection process.

### Serum depletion has limited influence of synchronous gene expression

**Figure 5:**
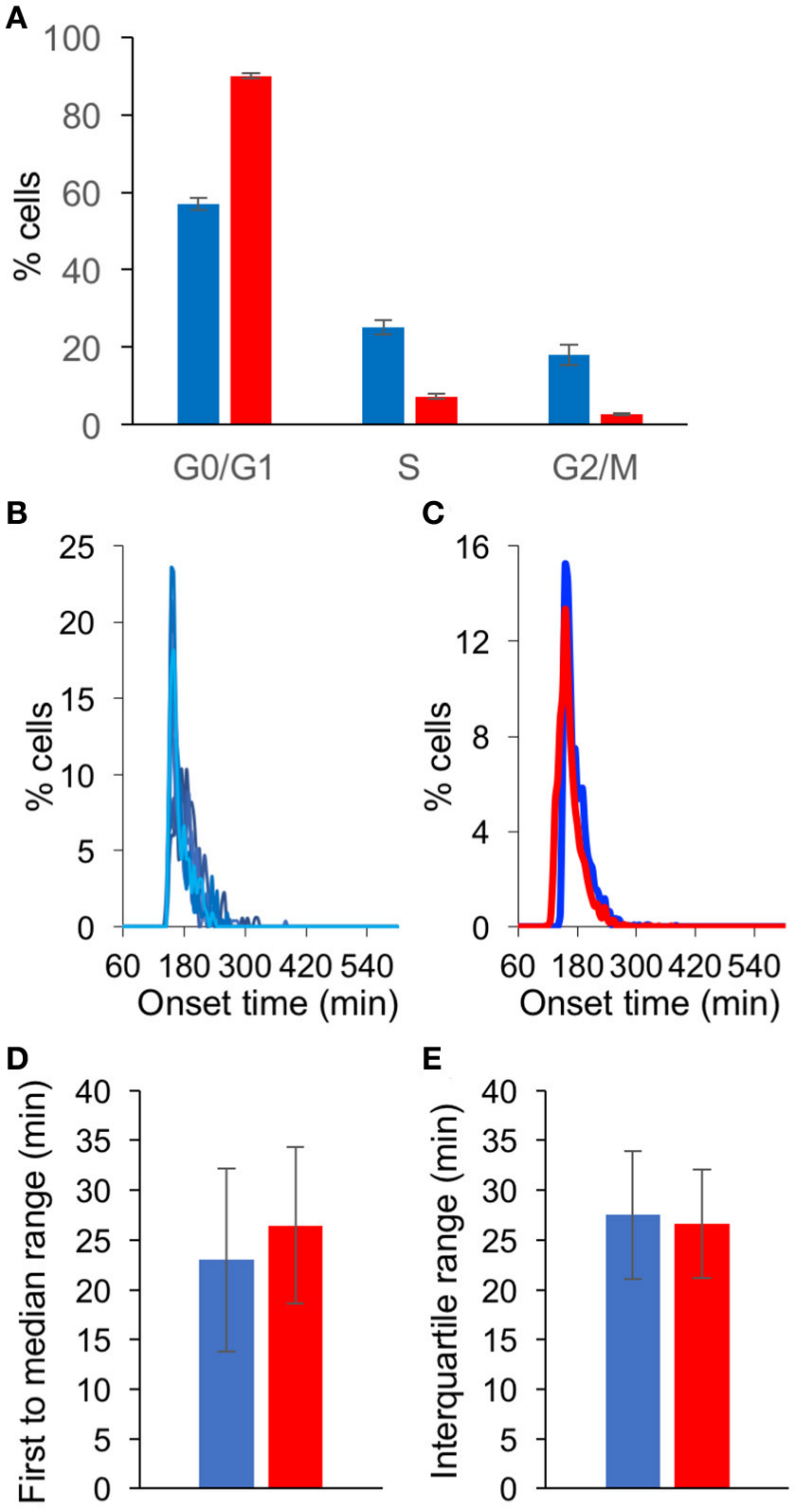
Serum depletion has limited influence of synchronous gene expression. **(A)** Cells were grown in 10% FBS for 24 h (blue) or in 0.5% FBS for 48 h (red). The cells were fixed, labeled with propidium iodide and analyzed to determine the cell cycle state in three replicative experiments. The percentage of cells at each cell cycle stage are presented. Error bars represent standard error among three plates (see Supplementary Figure 2). **(B)** Cells grown in 10% FBS were infected with OK11 recombinant virus at 39°C, at MOI 20. Histograms present the percentage of cells at each time point, that initiated expression (binning every 5 min). Eight wells from two experiments done at different days are plotted (four wells per experiment). **(C–E)** A comparison between the onset times of expression for the infection of cells grown in 10% FBS for 24 h (blue, data from **(B)** and cells grown in 0.5% FBS for 48 h (red, data from Figure 4A). **(C)** The average histograms for each condition were plotted. **(D)** The time between the first cell onset of expression to the median cell onset (first to median range) was plotted for each condition. **(E)** The interquartile range was plotted for each condition. **(C–E)** Error bars represent the standard deviation among the different wells.

To assess the contribution of serum depletion (a common practice to achieve cell cycle G0/G1 arrest, as was done in all the experiments above) to the simultaneous viral gene expression, we compared growing cells (un-arrested) and cells grown in serum depleted medium for 48 h (G0/G1 arrested). First, we tested the cell cycle state under these two conditions. As expected, we found that of the un-arrested cells, ~57% were at G0/G1 and ~18% at G2/M, compared to ~90 and ~3% for the G0/G1 arrested cells (Figure 5A and Supplementary Figure 2). We infected un-arrested cells at 39°C and monitored the onset of gene expression from these cells (Figure 5B). A comparison of the results obtained with the un-arrested and G0/G1 arrested cells (Figure 4A) resulted in very similar histograms (Figure 5C). Furthermore, no significant difference was observed between the two conditions, neither at the first to median range nor at the interquartile range (Figures 5D,E, respectively). We conclude that at elevated temperature and high MOI, the contribution of cell arrest to simultaneous viral gene expression is negligible.

### Elevated temperature reduces cell-to-cell variability in late stages of viral infection

**Figure 6:**
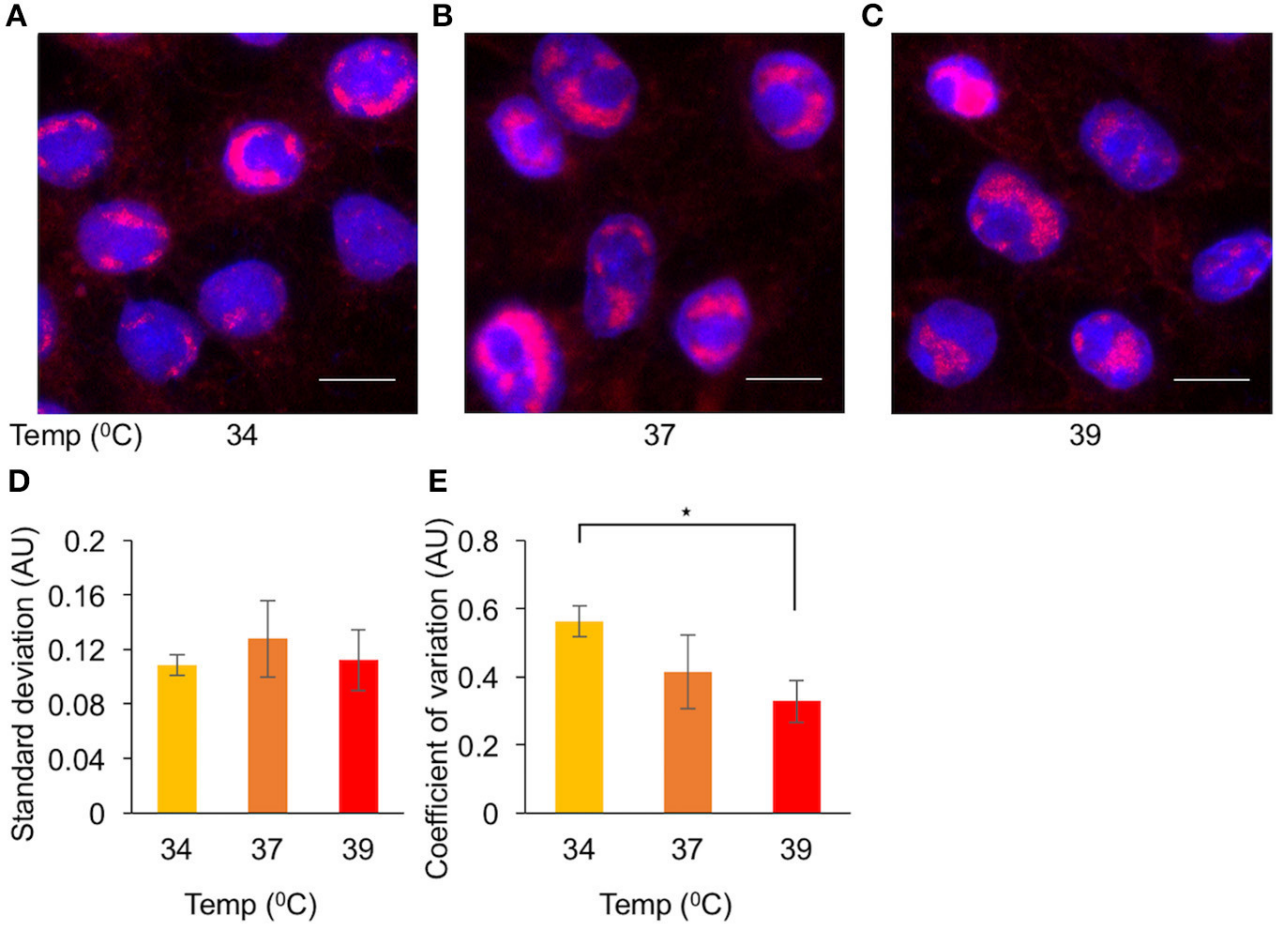
Elevated temperature reduces cell-to-cell variability in replication compartments size. Cells were infected with OK11 recombinant virus at MOI 20. Four HPI the cells were fixed and processed for FISH. Representative images of infection at 34°C **(A)**, 37°C **(B)**, and 39°C **(C)** are shown. Nuclei are colored with Hoechst stain in blue, viral DNA in red. Scale bar 10 μm. **(D,E)** A noise comparison between the relative size of viral RCs at different temperatures, (as indicated) was performed. **(D)** The standard deviation and **(E)** the coefficient of variation (standard deviation divided by the mean) were compared. Error bars represent the standard error of the means among the different frames. ^*^P < 0.05 by student *T*-test.

We found the elevated temperature has the largest impact on simultaneous viral gene expression (Figure 4). To test whether the elevated temperature will also reduce the diversity among cells in other parameters of the viral replication cycle, we decided to visualize the size of replication compartments (RCs) and to measure variability under different conditions. We infected cells at MOI 20 at different temperatures: 34, 37, and 39°C. At 4HPI, the cells were fixed, and the RCs were visualized by fluorescent *in situ* hybridization (FISH, Figures 6A–C). To determine the cell-to-cell diversity of the size of the RCs, we measured, for each cell, the total area of all RCs together. To account for the variability of the cells, for each cell, we divided the total area of all the RCs by the size of the nucleus. The ratio of the RC area to the nucleus area for each cell was used to quantify the variability among cells. While the standard deviation of this parameter did not change at the different temperatures (Figure 6D), the coefficient of variation (Raser and O'Shea, 2005) (the standard deviation divided by the average size), decreased at the higher temperatures (Figure 6E). Our results suggest that the diversity in the size of RCs, which probably represents the amount of viral replication that occurred in each cell, can be reduced by carrying out infection at 39°C. We speculate that this reduction may be attributed to the simultaneous onset of viral gene expression.

**Figure 7:**
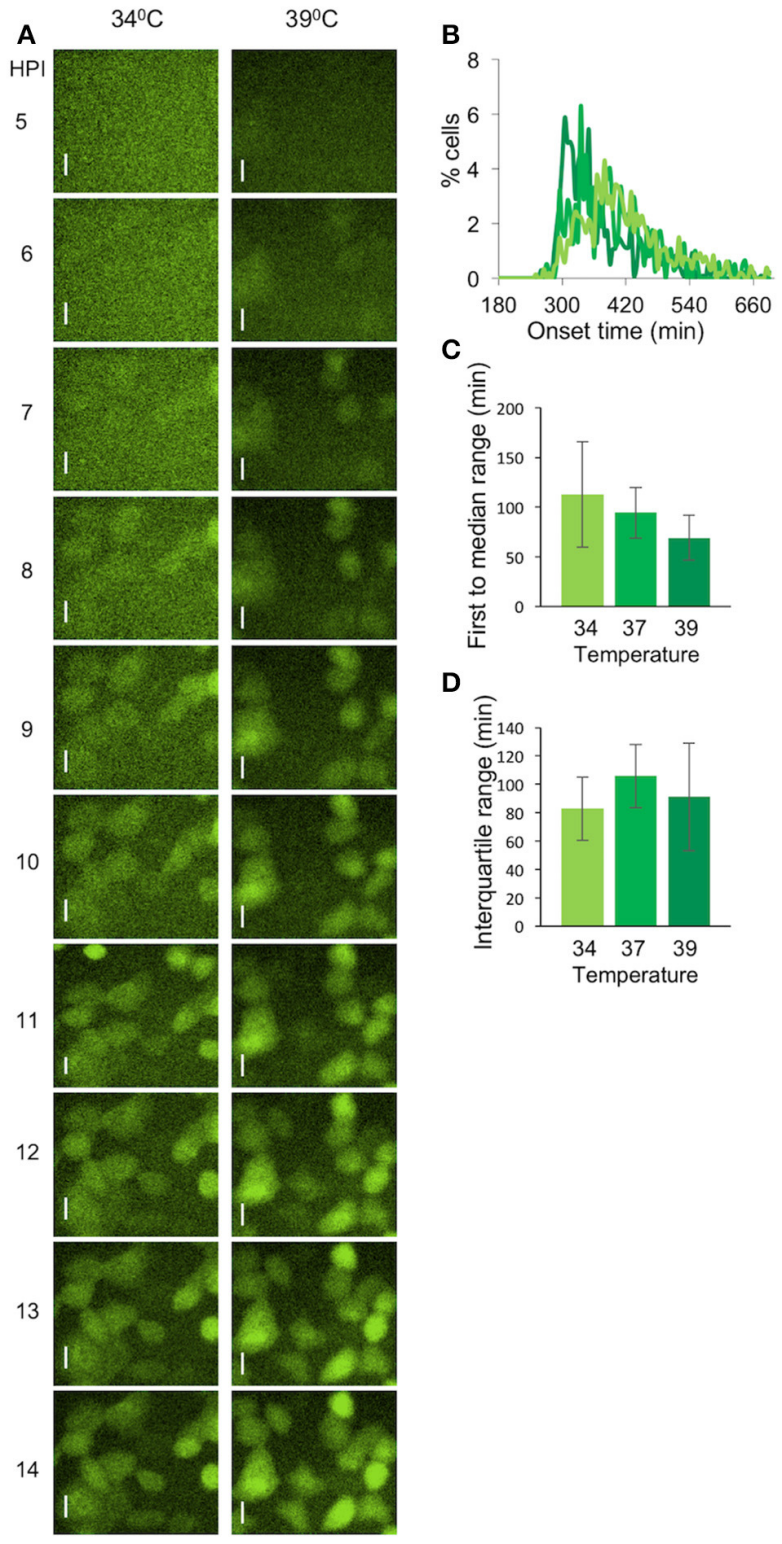
Variability of viral late gene expression exhibit temperature dependent. **(A)** Cells were infected with OK19 recombinant virus at MOI 20 and incubated at either 34°C (left) or 39°C (right). Snapshots at different time points (as indicated) are presented. mVenus fluorescence in green. Scale bar 20 μm. **(B–D)** A comparison between the onset times of expression for the infection at different temperatures: 34°C (light green), 37°C (green), and 39°C (dark green). **(B)** The average histograms for each condition were plotted. six wells from two experiments done on different days were plotted **(C)** The time between the first cell onset of expression to the median cell onset (first to median range) was plotted for each condition. **(D)** The interquartile range was plotted for each condition. **(B–D)** Error bars represent the standard deviation among the different wells.

To test whether the elevated temperature will also reduce the diversity of viral late gene expression, we infected cells at the different temperatures with OK19 strain. OK19 is a strain that carries the mVenus fluorescent gene within the UL25 gene and is expressed with the protein from the native late viral promoter. The onset of late gene expression is much later and nosier compare to the expression from OK11 (Compare Figure 7A to Figures 4E,F). However, differences between the temperature can still be observed. Figure 7A shows that infection at 39°C is faster and appear more synchronous that in 34°C. A comparison of the histogram obtained from infection at 34, 37,_,_ and 39°C show this trend (Figure 7B). However, due to the much nosier expression of the late genes, no significant difference were observed between the three conditions, neither at the first to median range nor at the interquartile range. We note that the first to median range does show the temperature dependent trend although not significantly. These results further indicate the importance of temperature in synchronizing the infection.

## Discussion

Here, we revisited the concept of synchronous viral infection in the case of herpes simplex virus. We started from the common protocols used to achieve synchronous viral infection and found that when observing single cells these protocols result in substantial variation. We tested several ways to increase simultaneous viral gene expression and found that increasing temperature and MOI reduced variability. Thus, in our experimental system, the most synchronous infection observed was with a wild type virus (OK11) at MOI 20 and incubated at 39°C. Synchronizing the nuclear entry or arresting the cells at G0/G1 had little or no effect on the simultaneous viral gene expression. By using the new conditions for synchronous viral gene expression, we were able to reduce coefficient of variation among cells of the viral RCs size.

What are the advantages of synchronizing viral infection? With the current state of the art technology, population based biochemical assays can provide detailed characterization of viral host interactions that currently cannot be obtained by single cell assays (for example; Dembowski and DeLuca, 2015; Kulej et al., 2017). However, viral host interactions during the lytic infection are highly dynamic (Lacasse and Schang, 2012; Lee et al., 2016) and better synchronization of the infection process, would allow a more detailed time resolution analysis of these interactions.

We used OK11 and OK24 viral recombinants in our experiments. Both recombinants contain the mCherry–NLS gene under the immediate early CMV promoter at the same site in the viral genome (between UL37 and UL38). We used this construct as we assumed it has the least effect on the expression of the other viral genes. The expression from this construct was found to correlate with native viral gene expression (Cohen and Kobiler, 2016) and a similar construct (mTurq2 instead of mCherry) was shown to have a similar onset to viral immediate early genes (ICP4, Drayman et al., 2017).

The CMV promoter was found to be induced by hyperthermia (Kobelt et al., 2010; Pshenichkin et al., 2011). Thus, our results from the CMV promoter may be in part due to the promoter and not only due to the virus regulation. Our results that reduced noise can be detected in later stages of the viral replication process (Figures 6, 7) provide evidence that the temperature dependent reduction in gene expression variability (Figures 3, 4) is due to the viral regulatory mechanism.

We found that increasing the MOI from 5 to 20 reduces the variability in the timing of viral gene expression among cells (Figure 2). At MOI 5, assuming Poisson distribution, 99.3% of the cells are infected with one or more viral plaque forming units (PFUs). As HSV-1 (like most other mammalian viruses) PFUs usually represent more than 10 viral particles (Flint et al., 2015), it is reasonable to assume that at MOI 5, each cell is exposed to dozens of viral particles. Although many viral particles enter the cell even at MOI 5, our results show that increasing this number can provide better synchrony. This can result from the very limited number of viral genomes that initiate expression in each cell, as we have previously characterized (Kobiler et al., 2010; Taylor et al., 2012; Shapira et al., 2016). We have shown recently that the number of viral genomes initiating replication in an individual cell correlates with the viral gene expression levels (Cohen and Kobiler, 2016). While the onset of expression is not always indicative of the levels of expression, there is a correlation between the two parameters (Supplementary Figure 3). Thus, the onset of gene expression may be influenced by the number of viral genomes initiating replication. As at MOI 5 this average is ~3.5 (Taylor et al., 2012), and in MOI 20 it is probably higher, we speculate that variation from the average at the lower MOI may result in a more significant variability in the onset of expression.

The well-documented tsB7 mutant (Knipe et al., 1981; Batterson et al., 1983; Jovasevic et al., 2008; Abaitua et al., 2009, 2011) is unable to enter the nucleus at the NPT. It was suggested that this mutant can dock to the NPC but is unable to release the viral DNA into the nucleus (Jovasevic et al., 2008). Our results with OK24, which is a derivative of the tsB7 mutant, show that only 2 min at the PT are sufficient for nuclear entry (Figures 3A,B). This finding suggest a rapid conformational change in the tegument protein VP1/2 which is sufficient to allow opening of the viral capsid and DNA release. Furthermore, this corroborates the finding that the injection of the viral genome through the nuclear pore into the nucleus is a very rapid process (Meyring-Wosten et al., 2014). While our results indicate that the nuclear entry can be tightly synchronized, there is no improvement in gene expression synchrony (compared to OK11 infection, Figure 3) under these conditions. These results suggest that most of the variability of herpes gene expression among cells is a consequence of viral host interactions within the nucleus. This variability can result from the many intrinsic antiviral mechanisms (located in the nucleus) which are able to inhibit viral replication (recently reviewed in Komatsu et al., 2016). Alternatively, this variability may result from the stochastic viral host interactions, such as the chance that the viral VP16 complex with the cellular proteins, will find viral genomes within the nucleus and activate the viral expression cascade (Kutluay and Triezenberg, 2009; Hancock et al., 2010; El Bilali et al., 2017).

The effect of temperature on variability among cells is striking (Figure 4). Small variations in temperature can have a major effect on heterogeneity among cells in viral gene expression. In our preliminary experiments, we found inconsistent results if the temperature and temperature shift were not tightly controlled. It is estimated that the noise in gene expression results from the stochastic nature of the many underlying biochemical reactions (Raser and O'Shea, 2005; Snijder and Pelkmans, 2011). These biochemical reaction events are in most cases enhanced by high temperature, including the rate of random diffusion. Theoretically, the higher temperature should therefore increase both the likelihood and rate of the viral gene cascade as well as the host antiviral gene activity. Thus, we speculate that under our experimental conditions (MOI 20) at the higher temperature where the viral gene expression is faster and more synchronized, the balance between viral expression program and the host antiviral mechanisms shifts toward the viral side.

The robust infection observed in temperature higher than the physiological temperature may result in unexpected and non-natural effects on the infection process. This might be the outcome of changes in the gene expression profiles of the host cells at the different temperatures (Schena et al., 1996). Thus, it is likely that several antiviral host proteins will change their expression patterns and therefore result in a change in the infection process. However, Herpes labialis is also known as “cold sore” as these blisters tend to develop during fever. Thus, it is possible that the common physiological temperature for the viral lytic process is higher than 37°C, raising the possibility that 39°C may better mimic natural conditions of the viral lytic infection. On the other hand, HSV-1 can be shed without cold sores (Ramchandani et al., 2016) and cold sores might develop without fever, suggesting that many new infections result from non-fever related conditions. Thus, we conclude that more studies are required to decipher the complex role of temperature on viral host interactions during herpes virus infection.

We have recently identified that the probability of HSV-1 infection as well as the kinetics of the infection process are dependent on the cell cycle (Drayman et al., 2017). Significant cell cycle effect was not detected in earlier studies using population based assays (Cohen et al., 1971; Cai and Schaffer, 1991). In this study, we could not detect an effect of arresting the cells at G0/G1 on the simultaneous viral gene expression or on the rate of infection progression. Each work was carried out using different cell lines, here we tested Vero cells compared to H1229 cells (human non-small cell lung carcinoma cell-line) in Drayman et al. Further, the methods for arresting the cell cycle were different between the studies, such that the Drayman et al. compared early S to G2/M cells whereas here we compared G0/G1 enriched to non-synchronized cells. Another key difference in this work compared to our previous work (Drayman et al., 2017) was the MOI used in the infections. In our work (as well as in the Cohen et al. paper), the MOI used was high compared to the Drayman et al. work done at MOI 0.5. We speculate that when a high MOI is used, the viruses are able to overcome many differences emerging from the cell state.

Most HSV-1 RCs initiate from a single infecting genome (Sourvinos and Everett, 2002; Kobiler et al., 2011). They later move toward each other and coalesce (Chang et al., 2011). RCs grow in size as viral replication progresses. Here, we measured the proportional area of the RCs from the nuclear area as a parameter for viral DNA replication. Our results show that the variation decreases as the temperature increases (Figure 6). We therefore hypothesize that synchronization of the viral gene expression reduces cell-to-cell variability in other elements of the infection process.

To conclude, we investigated mechanisms for achieving simultaneous viral gene expression at the single cell level. We found that the timing of expression from the viral genomes varies among cells, and may contribute to the significant heterogeneity in the outcome of infection, observed among genetically identical cells. Simple modifications in the experimental process, like increasing MOI, and temperature, can substantially reduce this variation. These results suggest that single-cell experiments should be carried out for other viruses to identify the specific conditions for synchronized infection. We suggest that using the modifications in the experimental process we identified, in population-based biochemical and genetic experiments can reduce the effect of variability among cells.

## Methods

### Viruses and cells

Green monkey kidney cells (Vero) were grown with Dulbecco's Modified Eagle Medium (DMEM X1; Gibco), supplemented with 10% Fetal Bovine Serum (FBS; Gibco) and 1% Penicillin (10,000 units/ml) and Streptomycin (10 mg/ml) (Biological Industries Israel).

Viral recombinants OK11 and OK24 carry a single fluorescent protein (mCherry) with a nuclear localization tag under the CMV promoter between the UL37 and UL38 genes, as described previously (Taylor et al., 2012). OK11 is a derivative of the HSV-1 strain17+. OK19 is a derivative of the HSV-1 strain17+ containing the mVenus gene fused in frame within the UL25 gene after the 50th amino acid as described (Cohen and Kobiler, 2016). OK24 is a derivative of a homologous recombination between OK11 and the HSV-1 strain KOS/HFEM with a ts single point mutation mapped to the UL36 gene encoding VP1/2 with a Y1453H change (as described in Abaitua et al., 2011). The parental KOS/HFEM ts mutant was kindly provided by P. O'Hare (Imperial College, London).

### Time lapse image acquisition

Eight well-chambered coverglasses (Nunc™ Lab-Tek™ II 155411) were seeded with 1 × 10^4^ Vero cells per well and incubated overnight (DMEM X1 with 10% FBS and 1% Pen-Strep). For most experiments, the serum was replaced with a serum depleted medium (DMEM X1 with 0.1% FBS and 1% Pen-Strep) for 48 h. For unsynchronized cells, the infections were done immediately after the first night of incubation with the rich medium. The cells were inoculated with 100 μl of the specific virus at indicated MOIs for 1 h at 4°C (rocking the plate every 15 min). After inoculation, the cells were washed once with cold Phosphate buffered saline (PBS X1; Biological Industries Israel). For each experimental temperature, a 3°C warmer medium (DMEM X1 with 10% FBS, 1% Pen-Strep and 2.5 μg/ml Hoechst 33342; Invitrogen) was added (for example, for the 34°C experiments, a 37°C medium was added). To prevent overheating of the cells for the 39°C experiments, first, 50 μl of ice-cold medium were added to the cells and immediately after that, 200 μl of 42°C medium were added. The cells were placed in an incubator for at least 1 h before imaging was started.

Images were acquired using a Nikon Eclipse Ti-E epifluorescence inverted microscope every 10 min at the indicated temperatures in a 5% (vol/vol) CO_2_-enriched atmosphere using a Chamlide TC stage top incubator system (Live Cell Instrument).

For the temperature shift experiments, cells were incubated after inoculation in 250 μl medium for 1 h at 39°C (as described above). After 1 h, the slide was placed on a preheated (to 34°C) metal block in a 34°C incubator for either 2 min or for the rest of the experiment. For shifting back to the high temperature, 100 μl of medium at 43°C was added immediately before the slide was placed on a preheated (to 39°C) metal block in a 39°C incubator.

### Image analysis

Each cell was detected and tracked based on the nuclear stain (Hoechst 33342) using the Imaris8.1 (Bitplane) “spots” algorithm. The mean fluorescence level from the mCherry protein was obtained for each cell at each time point. We used a specific Matlab script to estimate the onset time for each cell. All codes used in this work are available upon request. Specifically, for each cell, we first determined the baseline fluorescent levels by averaging the first 10 points and plotting the horizontal line. In order to obtain the range of the initial onset, we determined the first time point (J) at which the fluorescent level was five standard deviations (calculated from the first five points) above the baseline. A polynomial of second degree was fitted to the time points from the first time point to 15 points after the J time point. The intersection between the baseline and the polynomial curve was determined as the onset point for each cell. An example is illustrated in Supplementary Figure 1. For each well, ~300 cells were analyzed and plotted (ranging from 52 to 1,030, with an average of 311 and a median of 294 cells per well).

### Quantitative PCR

Cells were infected under different temperature conditions, as indicated; temperature shifts were carried out in a similar way to the time lapse experiments. 16 HPI the cells were collected and incubated for 1 h with a lysis buffer containing 10 mM Tris-HCl, pH = 8.0, 1 mM EDTA (Merck), 1% Tween 20 (Sigma-Aldrich), 0.04% Proteinase K (BIO-LAB, Israel). Proteinase K was deactivated by exposure to 95°C for 10 min. Viral DNA was amplified using Faststart universal SYBR Green Master (Roche) in the presence of primers for a specific gene (UL19, see Cohen and Kobiler, 2016) and normalized to cellular genomes by PCR of the GAPDH gene (see Shapira et al., 2016). qPCR was carried out and analyzed in StepOne^TM^ (Applied Biosystems^TM^).

### Capsid protection assay

Cells were infected with the HSV-1 strain KOS/HFEM with a temperature sensitive Y1453H single point mutation at MOI 20 and collected at different points during the infection process. At the indicated times, the medium was removed and washed with ice cold PBS. To collect the cells, plates were scraped and the cell pellet was washed with ice cold PBS twice. After collecting the cells, the cells were kept on ice or at 4°C at all steps. The collected cells were resuspend in DNase I buffer with 0.1% NP40. The suspended cells were equally divided to two fresh tubes. To one of the tubes DNase was added and the cells were incubated for 48 h at 4°C with gentle mixing. After DNase incubation, the DNase was heat inactivated at 65°C for 10 min following with a capsid denaturation at 95°C for additional 10 min. The samples without the DNase were treated similarly in parallel. All samples were amplified using Fast SYBR Green Master Mix (Applied Biosystems^TM^) in the presence of primers for a specific gene (UL19, see Cohen and Kobiler, 2016). qPCR was carried out and analyzed in CFX96 real-time PCR system (Bio-Rad^TM^).

### Cell cycle analysis

2 × 10^6^ cells were plated in 10 cm plates and incubated overnight (DMEM X1 with 10% FBS and 1% Pen-Strep). The cells were either harvested after the overnight incubation or the serum was replaced with a serum depleted medium (DMEM X1 with 0.1% FBS and 1% Pen-Strep) for 48 h. Three plates from each condition were harvested and fixed with 70% ethanol for 48 h at −20°C. Samples were rehydrated with 0.1% Triton X-100 in PBS, treated with 10 μg/ml RNAse A (Sigma-Aldrich) and stained with 15 μg/ml propidium iodide (Sigma-Aldrich) to analyze the cell-cycle stage by Attune NxT cytometer (Life technologies).

### Fluorescent *in situ* hybridization (FISH)

Cells were infected under different temperature conditions, as indicated; temperature shifts were carried out in a similar way to the time lapse experiments. 4HPI the cells were fixed and permeabilized according to protocol (Cremer et al., 2008). Twenty probes conjugated to cy3 where designed to detect the CMV promoter-mCherry construct. Two- dimensional images were acquired using a Nikon Eclipse Ti-S epifluorescence inverted microscope with a 100X oil lens. To estimate the area of the RCs and nucleus, we used the “surfaces” algorithm of the Imaris8.1 (Bitplane) software. Four different randomly selected frames for each condition were analyzed; 12 cells were randomly selected and measured from each frame.

## Author contributions

MR and OK designed research; MR, MB, ET, DR, and SZ performed research; MR, MB, ET, SZ, and MG contributed new reagents/analytic tools; MR, MB, and OK analyzed data; and MR and OK wrote the paper.

### Conflict of interest statement

The authors declare that the research was conducted in the absence of any commercial or financial relationships that could be construed as a potential conflict of interest.

## Supplementary material

Supplementary Figure 1 Estimating the onset time of gene expression for each cell. The red fluorescence profile for individual cells during the infection was plotted in brown. The baseline calculated as a horizontal line by averaging the first 10 time points, was plotted in turquoise. The best fitting polynomial curve (order 2), was plotted in pink (see Methods for details). The intersection point between the curve and the baseline (indicated by an arrow to the circle) was considered the onset time for this cell.

Supplementary Figure 2 Cell cycle analysis of growing and arrested cells. Cells were grown in 10% FBS for 24 h (**right**) or in 0.1% FBS for 48 h (**left**). The cells were fixed, labeled with propidium iodide and FACS analyzed for fluorescence levels in three replicative experiments (one panel for each experiment). In each histogram, the number of cells was plotted as the function of the fluorescence from the propidium iodide in arbitrary units (AU).

Supplementary Figure 3 Correlation between fluorescence onset time and fluorescence levels. Cells were infected at MOI20 with OK11 recombinant virus and monitored for fluorescence levels every 10 min. The cells were divided into 10 groups according to the onset time. For each group the average onset time and average fluorescence level at 6 hpi were calculated and plotted. A trend line, calculated using the ordinary least squares (OLS) method and the measured Pearson correlation, is presented in the graph. Error bars represent the standard error of the mean for each group.

Supplementary Movie 1 Cells infected with OK11 at MOI 5 were monitored for 12 h at 37°C. Scale bar 20 μM. Images were taken every 10 min and played at a rate of three frames per second.

Supplementary Movies 2 and 3 Cells infected with OK11 at MOI 20 were monitored for 12 h at 34°C (2) or 39°C (3). Scale bar 20 μM. Images were taken every 10 min and played at a rate of three frames per second.
