# Supplementary figures and images for: Promoting Simultaneous Onset of Viral Gene Expression Among Cells Infected with Herpes Simplex Virus-1
Source: Front Microbiol. 2017 Nov 1;8:2152. doi: 10.3389/fmicb.2017.02152 (PMC5671993; doi:10.3389/fmicb.2017.02152)

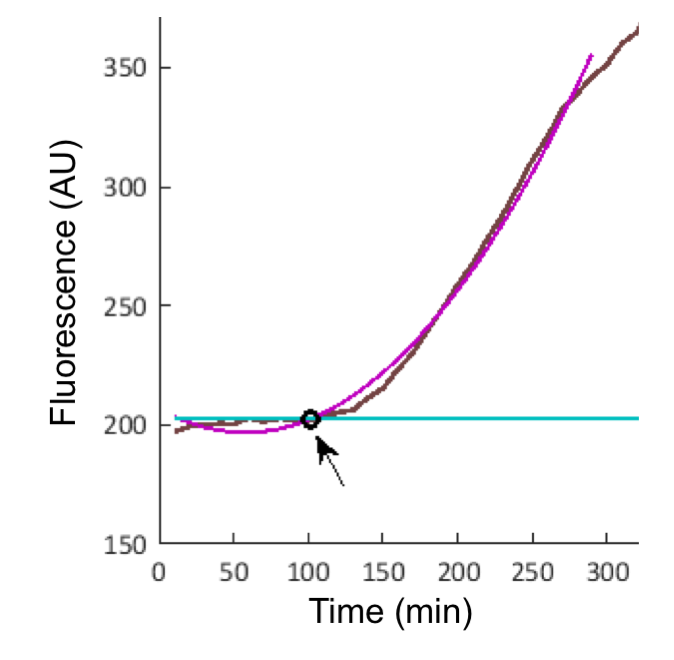

Supplement: Supplementary Figure 1 — Estimating the onset time of gene expression for each cell. The red fluorescence profile for individual cells during the infection was plotted in brown. The baseline calculated as a horizontal line by averaging the first 10 time points, was plotted in turquoise. The best fitting polynomial curve (order 2), was plotted in pink (see Methods for details). The intersection point between the curve and the baseline (indicated by an arrow to the circle) was considered the onset time for this cell. [file Image1.TIFF]

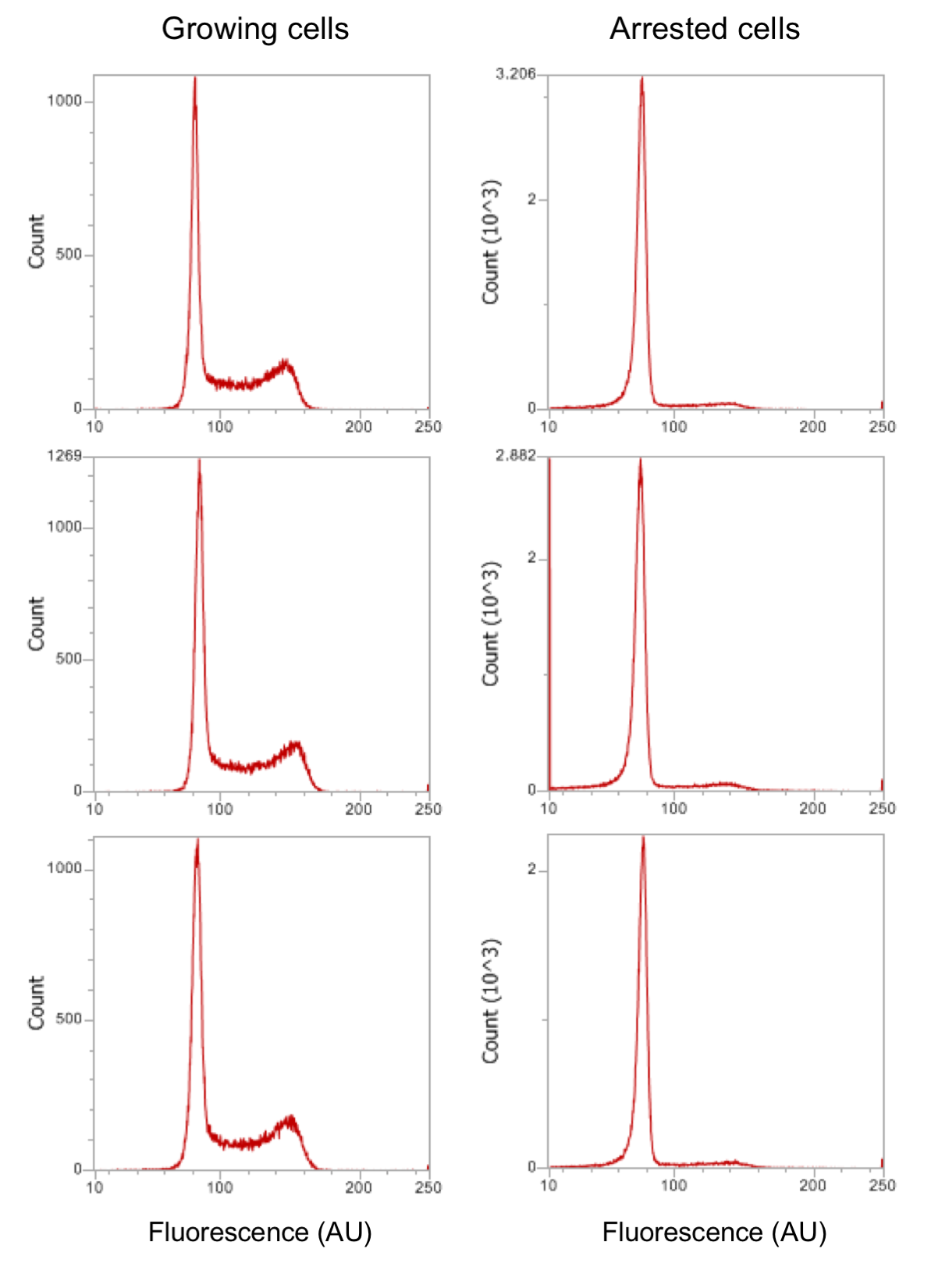

Supplement: Supplementary Figure 2 — Cell cycle analysis of growing and arrested cells. Cells were grown in 10% FBS for 24 h (right) or in 0.1% FBS for 48 h (left). The cells were fixed, labeled with propidium iodide and FACS analyzed for fluorescence levels in three replicative experiments (one panel for each experiment). In each histogram, the number of cells was plotted as the function of the fluorescence from the propidium iodide in arbitrary units (AU). [file Image2.TIFF]

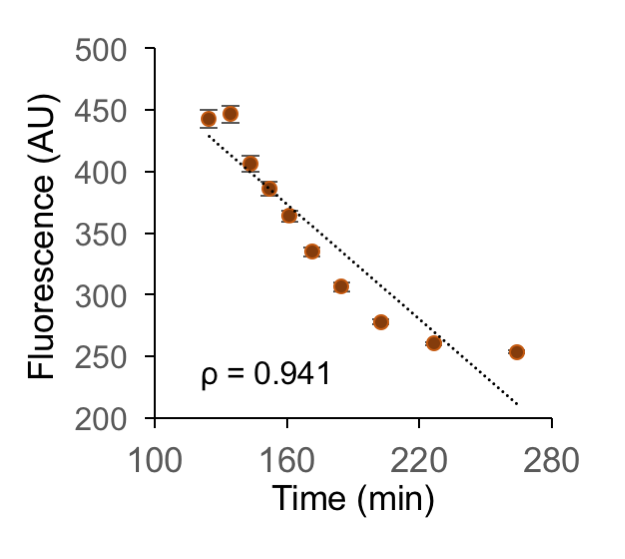

Supplement: Supplementary Figure 3 — Correlation between fluorescence onset time and fluorescence levels. Cells were infected at MOI20 with OK11 recombinant virus and monitored for fluorescence levels every 10 min. The cells were divided into 10 groups according to the onset time. For each group the average onset time and average fluorescence level at 6 hpi were calculated and plotted. A trend line, calculated using the ordinary least squares (OLS) method and the measured Pearson correlation, is presented in the graph. Error bars represent the standard error of the mean for each group. [file Image3.TIFF]
